# Primary caregivers’ practices and perceptions on antibiotic use and resistance: a one health qualitative study in rural South India

**DOI:** 10.1136/bmjopen-2025-112630

**Published:** 2026-05-12

**Authors:** Kamini Charan, Velappan Lakshmi Kandhan, R Sahaya Rishika, Palavesam Kalimuthu, A Charles Pon Ruban, Mohan Babu Karthikeyan, Krushna Chandra Sahoo, Manickam Ponnaiah, Girish Kumar Chethrapilly Purushothaman, Vishal Diwan

**Affiliations:** 1 Division of Environment Monitoring and Exposure Assessment (Water and Soil), ICMR-National Institute for Research in Environmental Health, Bhopal, India; 2 Department of Health Sciences, Savitribai Phule Pune University, Pune, India; 3 Model Rural Health Research Unit, Tirunelveli, India; 4 Tirunelveli Medical College, Tirunelveli, India; 5 ICMR-National Institute of Epidemiology, Chennai, India; 6 Department of Health Research, Ministry of Health and Family Welfare, New Delhi, India; 7 Faculty of Medical Sciences, Academy of Scientific and Innovative Research, Ghaziabad, India; 8 Department of Global Public Health, Karolinska Institutet, Stockholm, Sweden

**Keywords:** Public health, India, QUALITATIVE RESEARCH, Community child health, Antibiotics

## Abstract

**Abstract:**

**Objectives:**

Antimicrobial resistance is a growing global public health threat driven by interactions between human, animal and environmental factors. Rural settings in low- and middle-income countries may face increased risk due to unregulated antibiotic use, close human–animal interactions, and environmental contamination. This study explored community-level knowledge, attitudes and practices related to antibiotic use and resistance among caregivers of children in rural South India using a One Health perspective.

**Design:**

Qualitative study using focus group discussions and thematic analysis.

**Setting:**

Four rural villages in Tirunelveli district, Tamil Nadu, India.

**Participants:**

Seventy-seven primary caregivers of children aged 2–12 years from households with backyard animals, purposively selected from a rural cohort study.

**Results:**

Three themes emerged. First, human health practices included reliance on home remedies, reuse of prescriptions, self-medication and incomplete antibiotic courses alongside misconceptions about antibiotics. Second, environmental factors such as untreated water consumption, open defecation, poor drainage and improper waste disposal were perceived to increase infection risks. Third, animal-related pathways included close child–animal contact, antibiotic use in livestock and unsafe disposal of animal waste. Limited healthcare access and transport costs influenced treatment-seeking behaviour.

**Conclusions:**

Interconnected caregiver practices, environmental conditions and animal husbandry behaviours create multiple pathways for antimicrobial resistance transmission. Integrated, community-based interventions addressing behaviour change, healthcare access and environmental management are essential to support antibiotic stewardship within a One Health framework.

**STRENGTHS AND LIMITATIONS OF THIS STUDY**

- Focus group discussions with purposively selected caregivers provided contextual insights into antibiotic use practices in rural households.
- A semistructured guide and thematic analysis with independent coding strengthened methodological rigour.
- A One Health framework enabled exploration of human, animal and environmental factors related to antibiotic use.
- Findings rely on self-reported behaviours, which may be affected by recall or social desirability bias.
- The study was conducted in four villages within one district, which may limit transferability to other settings.

## Introduction

 Antimicrobial resistance (AMR) is widely recognised as one of the most serious global public health threats. Estimates from earlier global analyses suggested that drug-resistant infections cause at least 700 000 deaths annually worldwide, and without effective interventions this number could rise to 10 million deaths per year by 2050, potentially pushing 28 million people into extreme poverty due to its economic consequences.^1 2^ Antibiotics remain critical for reducing mortality, particularly among children; however, the global burden of AMR is substantial. A recent global analysis estimated that in 2019 nearly one in five deaths attributable to bacterial AMR occurred among children under 5 years of age.^3^ The emergence and spread of resistance are closely linked to increasing antibiotic consumption worldwide. Global antibiotic use increased by 36% between 2000 and 2010, with 76% of the increase attributable to Brazil, Russia, India, China and South Africa.^4^ More recent analyses show that antibiotic consumption has continued to rise globally between 2000 and 2015 and beyond, largely driven by increasing use in low- and middle-income countries (LMICs).^5 6^ India is among the largest consumers of antibiotics globally and faces significant challenges related to AMR.^4 7^

India is among the largest consumers of antibiotics globally and faces significant challenges related to AMR. Existing literature identifies several key drivers of AMR in India across human, animal and environmental sectors. Several interconnected factors contribute to the emergence and spread of resistance. Unregulated access to antibiotics, including over-the-counter (OTC) sales and dispensing without prescriptions by both licensed and informal providers, remains common due to limited healthcare access and weak regulatory enforcement.^8^,^12^ Antibiotic use in livestock and poultry production further contributes to the development and spread of resistance, with studies documenting resistant *Escherichia coli* and *Salmonella* isolates in food–animal systems.^13^,^24^ In addition, environmental contamination of water sources from untreated sewage, pharmaceutical waste and agricultural runoff creates reservoirs of antibiotic residues and resistance genes that facilitate transmission across bacterial populations and ecosystems, as demonstrated in studies documenting antibiotic residues and resistant bacteria in river water and sediments in India^25^,^30^

Children represent a particularly important population in the epidemiology of AMR.^3 7^ Frequent infections, higher exposure to antibiotics and close interactions with household environments increase the likelihood of colonisation and transmission of resistant organisms.^31 32^ In many rural settings in India, children regularly interact with backyard animals and contaminated environmental sources such as soil, water and waste disposal sites. These interactions create multiple pathways through which resistant bacteria may circulate between humans, animals and the environment within households and communities.^18 30 33 34^ Despite growing recognition of these interconnected pathways, most AMR investigations in India have focused on clinical or urban contexts and often examine individual sectors separately, thereby failing to capture the complexity of AMR transmission across human, animal and environmental domains.^20 35 36^

In addition to biological and environmental drivers, community-level behavioural factors play an important role in shaping antibiotic use and resistance.^7 35^ Caregiver knowledge, cultural health practices, health-seeking behaviour and household decision-making influence antibiotic procurement, adherence to treatment regimens and healthcare utilisation.^12 37^ Evidence from LMICs indicates that misconceptions about antibiotics, self-medication practices and incomplete adherence to prescribed treatment courses contribute significantly to the emergence and spread of resistance.^7 38^ However, these socio-behavioural determinants remain relatively underexplored in rural communities, where access to healthcare services is often limited and informal treatment practices are common.^12 39^

Recognising the interconnected nature of AMR across sectors, the One Health approach has been increasingly emphasised as a comprehensive framework for understanding and addressing AMR.^33 34^ The WHO Global Action Plan on AMR advocates integrated surveillance and coordinated interventions across human, animal and environmental domains.^3^ In India, the National Action Plan on Antimicrobial Resistance (NAP-AMR) further emphasises multisectoral collaboration to strengthen surveillance, stewardship and infection prevention across these sectors.^40^

Against this backdrop, the present qualitative study was conducted as a complementary component of a larger mixed-methods cohort study-One Health Epidemiological Study on AMR in Tirunelveli, South India (ONE-HEART) which aims to investigate the spectrum and distribution of AMR pathogens among children aged 2–12 years, backyard animals and environmental sources, including household waste and wastewater. While the quantitative component focuses on microbiological surveillance of AMR across human, animal and environmental samples, the qualitative component aims to contextualise these findings by exploring community-level knowledge, attitudes and practices related to antibiotic use and resistance among caregivers of children aged 2–12 years in rural households. Integrating microbiological and socio-behavioural insights provides a more comprehensive understanding of AMR transmission pathways within a One Health framework, thereby informing locally relevant prevention and stewardship strategies.

## Methods

### Study design and setting

This study employed a phenomenological qualitative approach to explore how caregivers interpret AMR based on their lived experiences.^41^ The study was conducted in rural areas of Tirunelveli district, Tamil Nadu, which comprises more than 370 villages grouped into nine subdistrict administrative units.^42^ The study was embedded within the Model Rural Health Research Unit (MRHRU), established by the Department of Health Research, Government of India, and mentored by the Indian Council of Medical Research–National Institute of Epidemiology (ICMR-NIE) in collaboration with the district government medical college and state public health system. The MRHRU maintains a population-based cohort of approximately 89 000 individuals across 26 villages. Within this platform, the ONE-HEART project was implemented to investigate AMR patterns in indicator organisms across human, animal and environmental interfaces and to examine antibiotic consumption practices. The study was conducted in four villages and included 165 children aged 2–12 years living in households with domestic animals, with additional sampling from livestock, poultry farms and the household environment to understand AMR dynamics in a One Health context.

### Study population

For the purpose of this qualitative study, we selected Primary Caregivers from the ONE-HEART study cohort, households were purposively sampled through preliminary interactions. ‘Primary caregiver’ was defined as a household with responsibilities of child care, animal rearing and overseeing household chores such as water storage and waste disposal. Eligible individuals were invited to participate in focus group discussions (FGDs). A total of 11 FGDs (seven female FGDs four male FGDs) were conducted, comprising 77 participants.

### Data collection

The research team included both male and female staff with backgrounds in public health, medicine, complementary medicine, environmental health, qualitative research, sociology and social work. The data collection team consisted of VLK, SR, KM and AC, all of whom had prior experience and training in qualitative research and interview techniques and were working as part of the project team within the institution. Two training sessions were conducted by VD and KCS to standardise procedures for conducting FGDs and note-taking.

FGDs were conducted using a semistructured interview guide consisting of open-ended questions covering awareness, attitudes and practices related to antibiotic use and procurement, waste disposal, water storage, sanitation facilities, hygiene practices among children and animal rearing. In addition, the guide explored indirect factors such as perceptions of healthcare systems and providers (see online supplemental file 1). The guide was translated from English into Tamil to ensure clarity and consistency across participant groups. The FGDs were guided by prompts covering key One Health domains, including children’s health-seeking practices, antibiotic use, animal contact and environmental exposures, and moderators used probing questions to ensure that all major domains were discussed during each session.

A pilot FGD was conducted with five female caregivers aged 23–27 years from the study area to assess the clarity and contextual appropriateness of the topic guide. Participants responded more readily to questions on children’s health-seeking practices, while responses to One Health and environmental aspects of AMR were limited. Based on these observations, the topic guide was revised to make questions related to environmental and One Health dimensions more general and accessible. As the participants met the study inclusion criteria and provided relevant information, data from the pilot FGD were included in the final analysis.

The research team-built rapport with potential participants during earlier visits and explained the study objectives. Individuals identified as eligible during the primary screening process were invited to participate in FGDs based on their willingness and availability. Government nursery schools (‘Balwadi’) or community halls were used as venues for the discussions. FGDs were conducted in Tamil by trained male and female moderators with assistance from a note-taker.

Prior to the discussions, demographic information was collected, and written informed consent was obtained from all participants for their participation as well as for audio recording of the discussions. Each participant was assigned a unique identification number to maintain confidentiality, with identifiers replaced by codes during transcription and analysis. Moderators introduced themselves and the note-taker, including their roles and the purpose of the study. Study objectives, participant rights and issues related to confidentiality were explained before the discussion began. The facilitators bracketed the preconceived notions, enabling participants’ experiences and viewpoints with an open mind, which further facilitated the emergence of themes that reflect the diverse experiences of caregivers across various demographics. No individuals other than the participants and the research team were present during the FGDs. Questions from participants were addressed during the session.

FGDs typically lasted approximately 45–50 min, although no fixed duration was predetermined, and discussions continued until key topics had been adequately explored. No repeat interviews or FGDs were conducted. Data collection continued until thematic saturation was reached, defined as the point at which no new themes or concepts emerged from successive FGDs. Saturation was assessed iteratively during data collection and preliminary analysis through regular discussions among the research team after each set of FGDs. Data collection was discontinued when additional discussions yielded no substantially new codes or thematic insights.^43 44^

### Data analysis

Each FGD audio recording was transcribed verbatim in Tamil and translated to English by SR, KD, MK and verified by a third-party translator to ensure contextual accuracy. Translations were then reviewed by KC, KCS, VD and GK, having prior qualitative analysis experience, for the purpose of data familiarisation and capturing overall depth and tone of the discussions. MAXQDA V.24 was used for data management and coding.

We performed a hybrid inductive-deductive analysis^45^ using the One Health framework. The data generated subcodes which were then organised into themes within the One Health domains, focusing on the human, animal and environmental domains. Furthermore, the codebook and themes were refined by independent coding, cross-checking and consensus among all authors. The analytic technique integrates inductive coding with a framework-based structure, using data-driven original and subcoding. Authors KC, KCS and VD identified data segments and developed subcodes, which were combined into interim codes. GK and PM contributed to coding for enhanced reflexivity. Two researchers established initial agreement through independent coding, followed by iterative revisions of a structured codebook. A third author resolved conflicts and finalised coding selections.

Codes were divided into categories based on their direct and substantial relationships. Where categories overlapped or varied by circumstance, they were divided into subthemes. This step specifically incorporates the One Health lens, interpreting topics based on the interconnectedness of human, animal and environmental health. Through iteration, the team developed three basic themes that unify One Health viewpoints throughout the three domains. These themes represent both the data-driven creation of concepts and their interpretation within the One Health paradigm. The study has been reported in accordance with the COREQ (Consolidated criteria for reporting qualitative research) checklist.^46^

## Results

### Profile of the participants

A total of 77 participants (47 females and 30 males) from diverse socioeconomic backgrounds were included in this study. The majority of them were aged between 20 and 39 years (n=43), with a few over 60 years. Educational levels ranged from illiterate (n=10) to secondary level of education, as most common (n=40). The majority of female participants reported being housewives (n=27), followed by farming (n=19). Monthly household income ranged widely, with the majority (n=56) reporting an income of up to ₹10 000 as stated in table 1.

**Table 1:** Socioeconomic profile of participants (n=77)

**Category**	**Subcategory**	**Frequency**
Age	20–39	43
	40–59	25
	60>	9
Occupation	Farmer	19
	Service/job	9
	Housewife	27
	Labour	19
	Unemployed	2
	Retired/pensioner	1
Education	Illiterate	10
	Primary	2
	Secondary	40
	Higher secondary	13
	Graduate	10
	Postgraduate	2
Monthly household income	<10 000	56
	10 000–30 000	19
	>30 000	2

### Thematic findings

**Figure 1:**
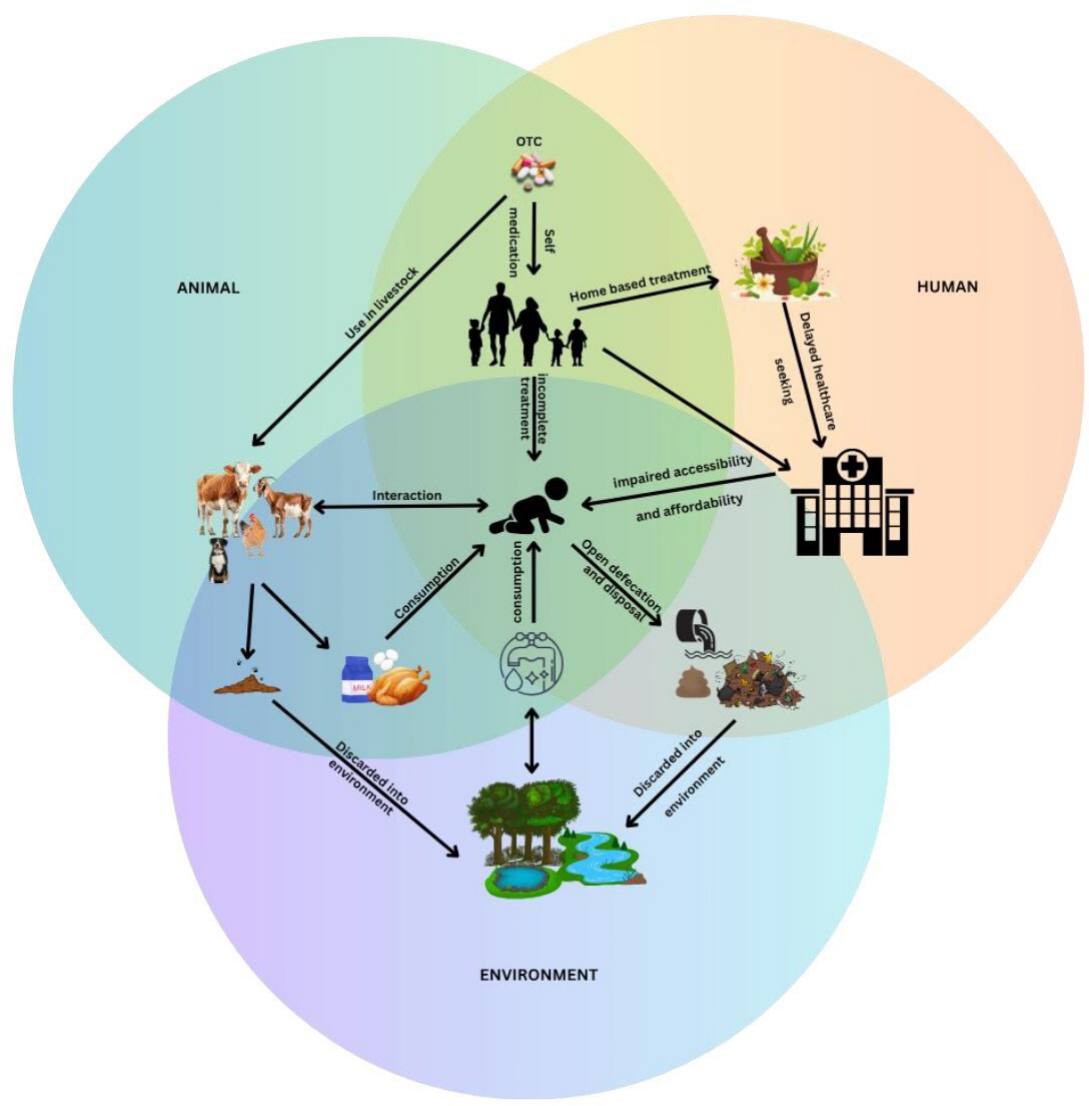
.Drivers of AMR among children in rural settings. AMR, antimicrobial resistance; OTC, over-the-counter sales.

Three overarching themes emerged, reflecting the interlinked domains of human, animal and environmental health (figure 1). These included the following: (1) awareness and practices affecting human health in a shared environment, (2) environmental AMR reservoirs affected by human activities and (3) animal mediated AMR pathways. These overarching themes consisted of multiple subthemes which have been discussed in detail through multiple categories as stated in table 2.

**Table 2:** Themes, subthemes and categories identified under thematic analysis

**Theme**	**Subtheme**	**Categories**
Awareness and practices affecting human health in a shared environment	Delayed seeking of appropriate healthcare	Common illnesses among children and perceived cause by caregivers
		Home remedies and spiritual healing practices as initial symptomatic treatment
		Stocking and reuse of medication and prescriptions
	Lack of awareness, understanding and non-adherence to antibiotic regimens	Antibiotics perceived as immune-boosting medicines
		Motivators and inhibitors towards treatment regimens completion
	Barriers in seeking timely and appropriate healthcare	Accessibility and affordability of healthcare as a challenge
		Perception towards healthcare system and providers leading to frequent change
Environmental AMR reservoirs affected by human activities	Water and sanitation infrastructure and practices	Type of water consumption and defecation practices followed by children
		Poorly maintained open drainage systems a breeding ground for infections
	Unavailability of timely waste collection leading to inappropriate disposal practices	Practices and facilities towards waste collection and disposal
		Open disposal and dumping of kitchen and medical waste
Animal mediated AMR pathways	Child–animal interaction and hygiene measures for infection prevention	Child-animal interaction and caregiver perception towards its risks
		Hygiene and preventive measures postinteraction with animal
	Practice of antibiotic use in animals, use of byproducts	Use of antibiotics in backyard animals
		Animal waste use and disposal

AMR, antimicrobial resistance.

### Theme 1: awareness and practices affecting human health in a shared environment

#### Subtheme 1: perceived cause of illnesses and delayed seeking of appropriate healthcare

##### Category: common illnesses among children and perceived cause by caregivers

Most participants reported fever, cough and cold as the most common illnesses affecting children. These were commonly linked to factors such as heat, playing in the rain and sudden changes in weather. Other mentioned conditions included headache, loose motions, stomach ache, skin allergies and vomiting. In addition to biological and ecological factors, some caregivers perceived mosquitoes from stagnant water canals as a reason for children falling sick.

If the main canal was clear, the places where children walk would be clean. We believe that if there were no stagnant water, there wouldn’t be mosquitoes, and there wouldn’t be fever or other illnesses. If the drainage system was proper and well-maintained, every household would be better off. (F-06, FGD 5)

##### Category: home remedies and spiritual healing practices as initial symptomatic treatment

A large number of participants consistently reported the use of home-based remedies as their first line of response when children showed signs of illness. These remedies typically included herbal concoctions prepared using ingredients such as ginger, tulsi (basil), pepper, papaya leaves, neem and betel leaves. These were widely used to treat common symptoms such as cough, cold and fever. For digestive problems, *Thokkanam*, a traditional therapeutic massage practice (also referred to as *Thokkam* or *Thokku* in the Siddha or Tamil medical system), was commonly used and was often perceived by caregivers to be more effective than allopathic medicines. Some caregivers reported giving goat’s milk and herbal decoctions such as kudineer and extracts of plants like thoothuvalai (purple fruited pea egg plant) and kandankathiri (yellow fruit nightshade).

I give them a mixture of dried ginger and pepper and let them drink it. If that doesn't help, we’ll take them to the hospital. If it’s severe, we’ll go to the hospital. (F-01, FGD 1)

We will take them to clear the blockage in the stomach (thokkam). If they have any symptoms like vomiting or diarrhea, we will take them to clear the blockage, and after that, everything will be fine. Other than this, we don’t seek any other treatments. (M-03, FGD 8)

Religious rituals were frequently mentioned, especially for treating symptoms such as fever and diarrhoea, which were often attributed to the ‘evil eye’. Rituals included tying sacred roots, sprinkling holy water and visiting temples or spiritual healers for blessings. Some caregivers believed that these practices gave them peace of mind in addition to the child’s recovery. However, a few participants, while aware of such practices, emphasised their preference for treatment at a hospital, citing a lack of faith in religious or spiritual interventions.

We will go to the priest and receive sacred ash. We also throw an egg to ward off the Drishti. (F-01, FGD 7)

My son once had a high fever and was very weak. We took him to Melapalayam (temple), where they tied the root and performed a ritual… After that, he got better. (F-04, FGD 6)

We don’t have faith in God, we trust the hospital only. (F-03, FGD 2)

##### Category: stocking and reuse of medication and prescriptions

Many participants reported keeping basic medicines such as paracetamol, cough syrup, tonics and previously prescribed tablets at home. These medications were typically used as the first response when a child falls sick, either alone or in combination with home remedies. Seeking medical care at a hospital was considered only if these initial efforts did not bring improvement. Another common practice was the reuse of old prescriptions to purchase medicines, particularly antibiotics.

I already buy and keep medicines at home. Since I stay at home, I always have medicines stocked. Whenever children have a fever or cold, I give them those medicines. (M-05, FGD 10)

They only tell you to use antibiotics for 5 days, and not more. So, we use the old prescription to buy it again. (F-03, FGD 3)

Seeking professional healthcare was frequently mentioned as a final option, usually after home remedies and available medicines failed.

If vomiting or something happens, we will try to settle it. After that, we will go to the temple to sprinkle water, only then we will go to the hospital. (F-01, FGD 5)

### Subtheme 2: lack of awareness, understanding and non-adherence to antibiotic regimens

#### Category: antibiotics perceived as immune-boosting medicines

Many participants admitted not knowing what antibiotics were, some considered them as immunity boosters, typically prescribed for fever and cold. A few participants equated turmeric, vaccines and Tetanus injections with antibiotics.

They stated that it is an immunity-boosting medicine and that its use will enhance strength. (F-06, FGD 4)

If someone gets a wound, they immediately apply turmeric powder that is an antibiotic. If the wound is more serious, they give a Tetanus (TT) injection that is also an antibiotic. (M-05, FGD 10)

#### Category: motivators and inhibitors towards treatment regimen completion

Most caregivers admitted not completing the full course of antibiotics or other prescribed medicines. The most commonly cited reasons included the child showing improvement within a few days, difficulty in administering medicines or side effects such as loose stools. Forgetting to continue the medication was also mentioned. Notably, one participant reported deliberately reducing the prescribed dose.

If the child is already better, why should we continue? It’s already hard enough to make them take medicine. (F-01, FGD 2)

If the doctor tells me to give the medicine for five days, I will give it for two days and then stop. (M-08, FGD 11)

Only a small proportion of participants reported completing the full course of prescribed medication, following motivation by medical advice or fear of internal illness and relapse.

The doctor said even if the fever subsides, you should continue because internal fever could be present. (F-02, FGD 4)

### Subtheme 3: barriers in seeking timely and appropriate healthcare

#### Category: accessibility and affordability of healthcare as a challenge

Distance and transportation costs were major concerns for most participants. Hospitals were far from their villages, making it difficult to access timely care, especially during emergencies at night. High auto fares and long travel times added to the hardship. Some participants spoke about spending an entire day on a hospital visit.

Even for an auto, they demand ₹500–600 rupees. (F-06, FGD 4)

Despite these challenges, many caregivers expressed their commitment to spend whatever was necessary for their child’s health.

No matter what, we’ll buy medicine. We don’t even look at the price. (M-01, FGD 10)

#### Category: perception towards healthcare system and providers leading to frequent change

Caregivers often changed doctors or hospitals if they felt a treatment was not effective or had greater trust in another provider. Government hospitals were often viewed with scepticism due to perceived inadequate care, limited treatment options, particularly the lack of injections.

In government hospitals, they don’t give injections for fever. That’s why more people go to private hospitals. (F-07, FGD 4)

Some participants followed a trial-and-error approach, trying multiple providers until they noticed some improvement.

We see different doctors. (F-04, FGD 4)

Currently, I am consulting three doctors. (F-05, FGD 7)

### Theme 2: environmental AMR reservoirs affected by human activities

#### Subtheme 1: water and sanitation infrastructure and practices

##### Category: type of water consumption and defecation practices followed by children

A large number of participants reported regular consumption of untreated water, often described as ‘normal’ or ‘raw’ water, reflecting a reliance on unfiltered sources for drinking. Water was obtained from multiple sources, including municipal taps, rivers and tanks. When asked about reasons for not consuming boiled water, one participant stated:

Because boiled water doesn’t taste the same. (F-03, FGD 4)

When asked about their drinking water source, one participant responded:

From the river and municipal tap water. (F-07, FGD 4)

Irregular water supply was stated as a recurring issue, resulting in participants storing water, leading to insect breeding and raising concerns about waterborne illness.

Since water does not come regularly, people store it. But if we store it for two days, insects start breeding, which is a health problem, right? Stagnant water easily attracts insects. (F-01, FGD 3)

For children, we are using only boiled and cooled-down water. (F-04, FGD 7)

While some children used household toilets, others defecated outdoors in riverbeds or nearby forests, particularly during the dry weather. In households with younger children, faeces were often wrapped in paper and disposed of outside the home or placed in nearby garbage bins.

The children do use it during the rainy season, but during other times, they go to the forest. (M-06, FGD 11)

We wrap it in paper and take it outside to throw it away. (F-03, FGD 5)

##### Category: poorly maintained open drainage systems a breeding ground for infections

Across all FGDs, participants consistently identified stagnant water in open drains as a breeding ground for mosquitoes, contributing to frequent fevers and infections in children. In some instances, children were reported to have fallen into open drains, underscoring the safety risks.

We have no proper drainage, no canals anywhere. The water from bathing, washing dishes, and other things, all goes to the front of the house. That’s why fever and infection spread. It’s the mosquitoes causing the problem, and it’s not going to improve. (F-06, FGD 5)

Participants also highlighted issues with irregular cleaning and poor maintenance of drainage channels and recommended weekly cleaning.

The drainage should be cleaned properly so that all the dirty water flows through one place. That would improve sanitation a lot. Some places are just left without proper maintenance (M-04, FGD 11)

### Subtheme 2: unavailability of timely waste collection leading to inappropriate disposal practices

#### Category: practices and facilities towards waste collection and disposal

Most participants described waste management practices that involved combining all types of household waste, including plastic and organic matter and burning it. While some participants reported access to garbage bins and collection services from the local Panchayat, very few practised waste segregation.

We won’t do it like that. We will put everything together. (F-01, FGD 7)

Since we use a wood furnace stove for cooking, we burn plastic in it (M-02, FGD 9)

In many areas, waste was reportedly collected only once every few weeks, resulting in overflowing bins. In some cases, only a portion of the garbage was collected while the rest was dumped near water bodies or in open spaces.

There’s a large garbage bin placed next to our house. We fill it up, and even if it overflows, it needs to be disposed of there. Once every three weeks, they come and collect the waste. (F-01, FGD 5)

Half of it goes to the garbage bins, where the collection vehicle takes it away. The other half is dumped near the pond. (F-01, FGD 3)

#### Category: open disposal and dumping of kitchen and medical waste

Participants commonly reported disposing of kitchen waste and wastewater into open drains or surrounding areas. This included discharge into canals, sewage systems, gardens, fields or empty spaces adjacent to their homes. Some households practised composting or directed the wastewater towards plants and trees as a form of reuse.

As for the vegetable waste, we will turn it into compost. (M-04, FGD 8)

We have cattle, so we throw it in the cow shed. (F-01, FGD 5)

In our house, the wastewater from the bathroom goes into the field, and it’s spread around. (M-05, FGD 10)

Most participants discarded unused or expired medicines along with general household waste or disposed of them in far-off places. Others reported practices such as burying medicines, mixing them with soil or placing them near plants. Notably, one participant mentioned sharing unused medicines with others if they were within the expiry date.

When the medicine expires, we gather them together and throw them away in a far-off place. (F-03, FGD 5)

I put them in plant soil. (F-02, FGD 9)

### Theme 3: animal-mediated AMR pathways

#### Subtheme 1: child–animal interaction and hygiene measures for infection prevention

##### Category: child–animal interaction and caregiver perception towards its risks

Participants reported that children had regular contact with goats, cows, dogs and chickens and engaged in activities such as feeding, cleaning and general play. Older children were also entrusted with animal care responsibilities. Although most participants acknowledged the possibility of germs spreading from animals such as dogs and animal waste, participants generally did not believe such interactions to be harmful to children and rather beneficial for the child’s development and sense of responsibility.

No, there’s nothing like that. At home, we tell them not to play with the dog, but even if we tell them, they won’t listen. They climb on the dog and play. But no illness has ever come from having the dog. (F-05, FGD 2)

The children feed the goats, tie the cows, and give them water. It’s all the children’s responsibility. If we’re not around, they have to do it. (M-04, FGD 11)

However, one participant acknowledged that children are exposed to infections by playing in dirt near goats and cows.

Children are exposed to infectious diseases because they play in the dirt near goats and cows. (F-02, FGD 3)

Dogs have tiny fleas, which cause skin allergies. (F-02, FGD 6)

##### Category: hygiene and preventive measures postinteraction with animals

Several participants emphasised that they ensured that the children washed their hands after playing with animals, with some parents also changing clothes or giving the child a bath. A few participants resorted to restricting, limiting or preventing direct contact between young children and animals, particularly dogs, due to perceived risks of bites.

Yes, they will wash their hands. Only after washing their hands, will we feed them food, but they will eat chocolate and other things without washing their hands*.* (F-02, FDG 7)

I won’t let them play. I keep them inside with the doors closed. Having goats and cows brings mosquitoes and flies, increasing the risk of infections. (F-01, FGD 6)

### Subtheme 2: practice of antibiotic use in animals, use of byproduct

#### Category: use of antibiotics in backyard animals

Some participants acknowledged the use of antibiotics for their livestock, stating that the animals were given doses as they deemed necessary. A majority of participants reported providing milk from their own backyard animals, primarily cows and goats, to their children. Many participants emphasised that they relied on milk produced at home and avoided purchasing milk from external sources, indicating direct consumption of animal byproducts.

Since birth, we've only been giving milk from our cows. The child has been growing up with that milk. (F-01, FGD 5)

They give antibiotics to the calves. They give the necessary dose based on the need, that’s all. For the cows, they give the appropriate dose*.* (M-05, FGD 10)

#### Category: animal waste use and disposal

Most participants mentioned using waste from backyard animals, especially cow-dung, for plastering cow-sheds and front yards of their houses, making cow-dung fuel cakes and some even reported selling them. Some participants mentioned making compost from animal waste. Disposal practices varied, with some participants stating that animal waste was disposed of into a lake or pond near the house, while one participant had a separate waste disposal system for managing animal waste.

Nowadays, people throw everything into the lake or pond behind the house, cow dung and all waste in one place*.* (F-02, FGD 2)

We have a waste disposal system, we put it in three separate bins for waste, one for cow dung, and one for dog waste. (M-07, FGD 10)

### Discussion

In view of the renewed interest and importance regarding AMR and absence of studies in rural context, we carried out in-depth exploration among 77 primary caregivers selected from within a cohort set up for one health studies on AMR in a South Indian rural setting. By adopting a One Health approach, our qualitative inquiry not only captures human health behaviours but also illuminates the often-overlooked roles of animal husbandry practices and environmental contamination in driving AMR. The study showed a practical integration of how caregiver practices intersect human, animal and environmental contexts in rural settings, highlighting how these meanings influence antimicrobial use beyond the household.

The findings from our study identified delayed healthcare seeking and self-medication as practices shaping antibiotic consumption. In these communities, caregivers frequently resorted to home-based remedies and traditional therapeutic practices such as *Thokkanam*, a massage-based treatment associated with the Siddha (Tamil) medical system, for initial symptom relief, reflecting deeply rooted cultural practices. Spiritual beliefs also appeared to influence health decision-making in the management of common childhood illnesses such as fever and diarrhoea. Previous research from South India has similarly highlighted how culturally embedded health belief models shape caregivers’ responses to childhood illnesses and influence treatment choices.^47^ Such culturally informed health-seeking patterns may delay presentation to formal health facilities, thereby encouraging self-medication when symptoms persist.^38^ Alarmingly, participants described reusing previous prescriptions to procure antibiotics without medical consultation. This practice is strengthened by studies in both urban and rural Indian contexts, where OTC sales and informal distribution channels persist despite regulations.^9 10 14^ Furthermore, the findings explored the persistence of regulatory and distributional conditions, which enable the OTC sales of antibiotics. Self-medication with antibiotics is a key driver of AMR in India, recommending urgent educational and regulatory measures to curb this trend.^48^ The ease of access to antibiotics further compounded by profit-driven incentives among wholesalers and pharmacists creates a self-reinforcing cycle of misuse, incomplete treatment courses and increasing selection pressure for resistant pathogens.^8 10 14 20^

In our study, we found many caregivers lacked a clear understanding of what antibiotics are, as some believed antibiotics are immune-boosting or equivalent to traditional remedies. Such misconceptions parallel findings from other LMICs, where fundamental knowledge deficits about antibiotic indications and risks are widespread.^8 12^ For instance, in rural Nigeria, Isha *et al* found that caregivers often misclassified antibiotics as general medicines, leading to inappropriate usage patterns.^49^ Our findings highlight that non-adherence to prescribed antibiotic courses was common: caregivers frequently discontinued antibiotics once symptoms subsided, citing difficulties in administering, side effects (eg, loose stools) or perceived recovery. Kolberg *et al* have documented how such suboptimal adherence contributes to prolonged illness, higher healthcare costs and increased transmission of resistant organisms in paediatric populations.^50^ Although a few participants reported completion of the full course following motivation from trusted physicians, this was the exception rather than the norm. These behaviours underscore the urgent need for targeted, culturally appropriate educational campaigns that emphasise the rationale for completing prescribed courses and the pitfalls of premature discontinuation.^51^

Further structural barriers to healthcare access were found to exacerbate AMR risks through our study. Participants described logistical and financial challenges in accessing healthcare, including long-distance travel and high cost (US$6–7) to reach the government hospitals, discouraging them from seeking care. These observations align with several reviews of rural Indian healthcare, which highlight systemic poverty, underdeveloped infrastructure and distrust of public services as key contributors to underutilisation.^52^,^54^ High out-of-pocket expenses and perceptions of inadequate care in government facilities prompted caregivers to engage in ‘doctor shopping’ until they found a provider whose treatment yielded quick symptomatic relief.^55^ Such practices affect continuity of care resulting in overlapping, often redundant antibiotic prescriptions, thereby amplifying selection pressure for resistance.^55^ Centralised digital healthcare records can be utilised to identify and control such practices by leveraging robust Health Information Technology.^56^ Addressing these structural inequities will require investments in rural health infrastructure, improved staffing and reliable supply chains at primary health centres to reduce dependency on OTC antibiotic procurement.

Our findings highlight the widespread consumption of untreated or raw water from rivers and municipal taps. Additionally, open defecation during dry seasons, poorly maintained open drainage channels and irregular waste collection services that exacerbate environmental reservoirs of resistant bacteria were frequently reported. These conditions mirror patterns documented in other parts of rural India, where inadequate water, sanitation and hygiene infrastructure are recognised facilitators of AMR by creating continuous transmission cycles among humans, animals and the environment.^26 27 57^ Pruden *et al* have demonstrated how rural wastewater and surface water bodies contain antibiotic residues and resistance genes, serving as conduits for horizontal gene transfer among bacterial populations.^18 26^ The indiscriminate disposal of expired medicines and animal waste into fields, ponds or open drains further intensifies environmental contamination, fostering persistent reservoir of resistance that re-enter the human and animal food chains.^26 58^ Systematic improvements in sanitation, waste management and access to safe water, interventions targeting antibiotic stewardship play are crucial in AMR control.

The findings based on human–animal interactions and antibiotic use in livestock highlight zoonotic transmission pathways that often go unaddressed in conventional AMR studies. In these rural households, children routinely interacted with backyard animals without stringent hygiene practices, increasing the potential for microbial transmission.^13^ Caregivers reported administering antibiotics to their livestock. Several participants fed milk to children from animals recently treated with antibiotics, unaware of the risks posed by antibiotic residues or the transfer of resistant bacteria to humans. These household level practices reflect broader patterns in LMICs and other Indian states’ agricultural systems, where antibiotic use for disease prevention frequently occurs without veterinary oversight.^1358^,^62^ It has been emphasised that such agricultural antibiotic practices pose significant risks to human health, advocating for stringent stewardship and monitoring in food animals to reduce zoonotic transmission.^17 21 22^ Moreover, horizontal gene transfer of resistance determinants from animal faeces via soil or water to human-associated bacteria further underscores the complexity of AMR ecosystems.^18^ Our findings reinforce calls for integrated ‘One-Health’ surveillance that encompasses veterinary antibiotic consumption data, residue testing in animal products and genomic monitoring of resistance genes across human, animal and environmental samples.^13 26^ The One Health framework advocated by the WHO offers a coherent conceptual framework to understand these shared risks and coordinate multisectoral responses.^20 25 33^

### Strength and limitations

This study’s strength lies in its application of the *One Health framework*, capturing the interconnected human, animal and environmental dimensions of AMR transmission. By employing a qualitative design, the study generated in-depth, context-specific insights into behavioural, cultural and structural determinants of antibiotic use and misuse. As with most qualitative studies, reliance on self-reported behaviours may introduce recall or social desirability bias. However, the saturation of narratives across FGDs strengthens the credibility of the findings.

Data were collected from four villages within a single district in southern India; therefore, the generalisability of findings to other settings within the state or across India may be limited due to geographical and sociocultural variability. Data saturation was assessed at the thematic level, where no new codes or themes emerged from additional FGDs. However, saturation was assessed within the context of these four villages, and perspectives from other geographic regions may differ. Nevertheless, the breadth and depth of themes identified provide transferable insights into drivers of AMR, particularly in resource-constrained rural settings. The study primarily focused on community-level perspectives, which are central to public health interventions and may act as catalysts for cross-sectoral change, as community engagement can influence practices related to healthcare utilisation, antibiotic use and environmental management.

### Conclusions

The findings highlight how caregiver health-seeking behaviours, misconceptions about antibiotics, limited healthcare access and environmental and animal-related practices together shape AMR risks in rural settings. Delayed healthcare seeking, reuse of prescriptions and incomplete antibiotic courses reflect both behavioural and structural barriers to appropriate antibiotic use. Environmental conditions such as inadequate sanitation, unsafe water storage and poor waste management further contribute to potential transmission pathways, while close human–animal interactions and unsupervised antibiotic use in backyard livestock introduce additional zoonotic risks.

These findings suggest that AMR prevention strategies in rural settings should extend beyond clinical stewardship and incorporate community-level interventions addressing behavioural practices, healthcare accessibility and environmental management. Within a One Health framework, integrated efforts involving public health systems, veterinary services and local governance structures may help strengthen antibiotic stewardship, improve awareness and reduce environmental drivers of resistance. Given the qualitative design and the limited geographic scope of the study, further research across diverse rural contexts is needed to better inform policy and intervention development.

## Supplementary material

10.1136/bmjopen-2025-112630 online supplemental file 1

## Data Availability

Data are available upon reasonable request.
