# Supplementary material for: Primary caregivers’ practices and perceptions on antibiotic use and resistance: a one health qualitative study in rural South India
Source: BMJ Open. 2026 May 12;16(5):e112630. doi: 10.1136/bmjopen-2025-112630 (PMC13182428; doi:10.1136/bmjopen-2025-112630)
Supplement: online supplemental file 1 [file bmjopen-16-5-s001.docx]

**One Heart: One Health Epidemiological Study on Antimicrobial Resistance in Tirunelveli, South India**

**ICMR (NIREH, NIE, VCRI)**

**Topic/Discussion Guide: Caregiver**

**Date: / / Place:**

**Start time: End Time:**

**Moderator: Note Taker:**

**No. of Participants: FGD no.:**

**Sociodemographic details of Caregiver**

| Identification code | Age | Relationship with enrolled child | Occupation | Education | Household Income | House size | Name of the enrolled child | Age of the child |
| --- | --- | --- | --- | --- | --- | --- | --- | --- |
|  |  |  |  |  |  |  |  |  |
|  |  |  |  |  |  |  |  |  |
|  |  |  |  |  |  |  |  |  |
|  |  |  |  |  |  |  |  |  |
|  |  |  |  |  |  |  |  |  |
|  |  |  |  |  |  |  |  |  |
|  |  |  |  |  |  |  |  |  |
|  |  |  |  |  |  |  |  |  |

**Introduction**

This discussion aims to understand how caregivers (you) care for children when sick, which healthcare services are used, and why. Also to understand what factors influence your healthcare-seeking behaviour, understanding of antibiotics, and to know how children in your household interact with backyard animals. This information will help us identify the kind of awareness, common healthcare practices, and human-animal interaction in this region.

For your information this discussion will be tape recorded, transcribed verbatim and then analysed by team of researchers. The information recorded about you and the discussion will be kept confidential and will be deleted after the purpose of the study is achieved. You can withdraw from the study at any point of time.

- Do you have any question to what I have explained till now?
- Could you please sign the consent form?

Thank you so much for listening, understanding, and continuing with us for the discussion.

- Please introduce yourself by telling about your name, age, qualification, work status.

**Ground Rules:**

- Please listen attentively to what others are saying and contribute your thoughts respectfully.
- Feel free to share your honest opinions and experiences.
- There are no right or wrong answers.

***NOTE:**

A lot a number to each of the participants in the discussion to keep a track on order of their speaking while transcribing.

The participants of a group should be homogeneous **e.g. Caregiver of children aged 0-5 are a part of the same FGD group.**

**Note for Interviewer:**

- **Do not ask direct questions.**
- **The question should not be close ended.**
- **Ensure that enough probes are asked.**
- **Avoid leading questions/probe**

**1. Common Illnesses in Children**

Q1 What kind of illnesses is most common in your community among children?

(To other participants: Do you encounter some other illnesses as more common in children 2-5 and 5+ years of age in your area)

- How frequently do they occur?
- And what do you think cause these illnesses?
- Are there any seasonal patterns?

**2. Initial Treatment Approaches**

Q2. What kind of illnesses have your children for example had during the last few days/months/year?

- Please describe what did you do to your sick child?
- What type of treatment was preferred first
  - home remedies, traditional medicine (Siddha or Homeopathy), or allopathic treatments, leftover medicines from previous illnesses
- How effective do you find these treatments?
- Where did you take your child for treatment?
- How did you choose where to take your sick child for treatment?
- Was there ever a time when you wanted to go to some other facility for treatment but were not able to go? And why?
- What factors influence your decision of taking the treatment from a particular facility?

(Ask to all participants separately having different responses)

Q3. Do you always take treatment from same place every time your child falls sick? (Can you share an example?)

- If Yes, what are the reasons you chose to take treatment from this place or people?
- How does affordability and access affect your choice to take treatment from this place or people?

Q4. If No, Why did you change the place of treatment at that particular time or with respect to that particular illness?

- What things did you consider, to seek treatment elsewhere for that particular illness?
- Did logistical issues (like distance or wait times) contribute to your decision to switch providers?
- Did you seek out a provider who specializes in a particular type of illness for your child?
- Did you feel that the illness required more immediate or specialized care than what your usual provider offered?

Q5. Do you have any particular belief related to some illnesses when you prefer to take some specific treatment or go to some specific people or place for treatment or do not take any treatment at all? If Yes, Why? (Can you share an example?)

- For what specific illnesses do you prefer going to a specific people or place for treatment?
- Can you explain about these beliefs?
- Based on what do you have these beliefs?

Q6. Do you have any knowledge/ understanding about the treatment given to your child in different illnesses?

*(NOTE: If the participants mention of antibiotics ask the next question. Whereas if the participants are unaware, make a hypothetical scenario in order to explain them).

What do you understand about antibiotics? How do you think they work?

- Have you noticed anything specific about their packaging, like a red line?

Where do you usually get your medications?

- Doctor, Pharmacy with/without prescription

Has a doctor ever asked you to give complete the course of medicine given/prescribed to the child?

- Are children given the complete dose as said by the doctor? **If not, what were the reasons?**

Did the doctor recommend any tests?

- What tests were suggested? (Type of test)
- Were they conducted? **If not, what were the reasons?**
- Where did you have them done?
- What was the cost?
- What were the results?

**6. Waste Disposal and Sanitation**

What is the main source of drinking water for this area/village?

- Do you usually filter or boil it?

Can you explain about waste disposal methods of your household?

- Where dose wastewater from the kitchen goes?
- Is household garbage segregated?
- Where is household garbage disposed?
- What is done with leftover medications when they’re no longer needed? (Important probe)
- Does your household have a latrine?
- Do children here practice open defecation?
- Where do children defecate in the open?
- Where and how is it disposed

**Child-Animal Interactions**

How often do children interact with backyard/domestic animals?

- like feeding, cleaning, playing with the animal
- Are animal byproducts, in your child's diet? (From backyard animals)
  - Milk, Eggs or Others

Has your child ever got an infection due to an animal?

OR

Has the doctor ever told you that your child is sick due to an animal?

- What measures do you take to prevent your child from getting sick from animals?
- What do you think are the advantages and disadvantages of children interacting with backyard animals? (Frame this probe in context of animal and child health)

**8. Challenges and Suggestions for Improvement**

What challenges do you face as a caregiver when the child is unwell?

- Are there any challenges related do access and affordability?
- How effective do you find the treatment to?
- Do you have any suggestions on how healthcare services in this region could be improved?

**Notes/Additional information:**

**COREQ (COnsolidated criteria for REporting Qualitative research) Checklist**

A checklist of items that should be included in reports of qualitative research. You must report the page number in your manuscript where you consider each of the items listed in this checklist. If you have not included this information, either revise your manuscript accordingly before submitting or note N/A.

| **Sr. No.** | **Topic** | **Guide Questions/Description** | **Reported on**  **Page No** |
| --- | --- | --- | --- |
| **Domain 1: Research team and reflexivity** | | | |
| *Personal characteristics* | | | |
|  | Interviewer/Facilitator | Which author/s conducted the interview or focus group? | 8 |
|  | Credentials | What were the researcher’s credentials? E.g. PhD, MD | 8 |
|  | Occupation | What was their occupation at the time of the study? | 8 |
|  | Gender | Was the researcher male or female? | 8 |
|  | Experience and training | What experience or training did the researcher have? | 8 |
| *Relationship with*  *Participants* | | | |
|  | Relationship established | Was a relationship established prior to study commencement? | 8 |
|  | Participant knowledge of the interviewer | What did the participants know about the researcher? e.g. personal  goals, reasons for doing the research | 8-9 |
|  | Interviewer characteristics | What characteristics were reported about the inter viewer/facilitator?  e.g. Bias, assumptions, reasons and interests in the research topic | 8-9 |
| **Domain 2: Study design** | | | |
| *Theoretical framework* | | | |
|  | Methodological orientation  and Theory | What methodological orientation was stated to underpin the study? e.g. grounded theory, discourse analysis, ethnography, phenomenology,  content analysis | 7 |
| *Participant selection* | | | |
|  | Sampling | How were participants selected? e.g. purposive, convenience,  consecutive, snowball | 8 |
|  | Method of approach | How were participants approached? e.g. face-to-face, telephone, mail,  email | 8-9 |
|  | Sample Size | How many participants were in the study? | 9 |
|  | Non-participation | How many people refused to participate or dropped out? Reasons? | 9 |
| *Setting* | | | |
|  | Setting of data collection | Where was the data collected? e.g. home, clinic, workplace | 8-9 |
|  | Presence of nonparticipants | Was anyone else present besides the participants and researchers? | 8-9 |
|  | Description of sample | What are the important characteristics of the sample? e.g. demographic  data, date | 9-10 |
| *Data collection* | | | |
|  | Interview guide | Were questions, prompts, and guides provided by the authors? Was it pilot tested? | 9-10 |
|  | Repeat interviews | Were repeat interviews carried out? If yes, how many? | 9 |
|  | Audio/visual recordings | Did the research use audio or visual recording to collect the data? | 8-9 |
|  | Field notes | Were field notes made during and/or after the interview or focus group? | 9 |
|  | Duration | What was the duration of the interviews or focus group? | 9 |
|  | Data saturation | Was data saturation discussed? | 10 |
|  | Transcripts returned | Were transcripts returned to participants for comment and/or correction? | No |
| **Domain 3: analysis and findings** | | | |
| *Data Analysis* | | | |
|  | Number of data coders | How many data coders coded the data? | 9 |
|  | Description of the coding tree | Did authors provide a description of the coding tree? | 10-11 |
|  | Derivation of themes | Were themes identified in advance or derived from the data? | 10 |
|  | Software | What software, if applicable, was used to manage the data? | 9 |
|  | Participant checking | Did participants provide feedback on the findings? | No |
| *Reporting* | | | |
|  | Quotations represented | Were participant quotations presented to illustrate the themes/findings?  Was each quotation identified? e.g. participant number | Throughout the results |
|  | Data and findings consistent | Was there consistency between the data presented and the findings? | Yes |
|  | Clarity of major themes | Were major themes clearly presented in the findings? | Yes |
|  | Clarity of minor themes | Is there a description of diverse cases or discussion of minor themes? | Yes |

Developed from: Tong A, Sainsbury P, Craig J. Consolidated criteria for reporting qualitative research (COREQ): a 32-item checklist for interviews and focus groups. International Journal for Quality in Health Care. 2007. Volume 19, Number 6: pp. 349 – 357
