# Supplementary material for: Primary caregivers’ practices and perceptions on antibiotic use and resistance: a one health qualitative study in rural South India
Source: BMJ Open. 2026 May 12;16(5):e112630. doi: 10.1136/bmjopen-2025-112630 (PMC13182428; doi:10.1136/bmjopen-2025-112630)
Supplement: Supplementary file 2 [file bmjopen-2025-112630.reviewer_comments.pdf]

PEER REVIEW HISTORY

BMJ Open publishes all reviews undertaken for accepted manuscripts. Reviewers are asked to complete a checklist review form and are provided with free text boxes to elaborate on their assessment. These free text comments are reproduced below.

ARTICLE DETAILS

Title (Provisional)

Primary Caregivers’ Practices and Perceptions on Antibiotic Use and Resistance: A One Health Qualitative Study in Rural South India

Authors

Charan, Kamini; Lakshmi Kanthan, V; Rishika, Sahaya; Kalimuthu, P; Pon Ruban, Charles; Karthikeyan, M; Sahoo, Krushna Chandra Chandra; Ponnaiah, Manickam; Girish Kumar, C.P.; Diwan, Vishal

---

VERSION 1 - REVIEW

**Reviewer**	**1**
**Name**	**Ancillotti, Mirko**
**Affiliation**	**Uppsala University**
**Date**	**26-Jan-2026**
**COI**	**None**

General comments

I am thankful to the editors and the authors for the opportunity to read and comment on this manuscript. The study design and data collection appear broadly appropriate, and the empirical material has the potential to make a valuable contribution to the literature. However, in my opinion, some issues need to be addressed before the manuscript can be considered for publication.

There are substantial problems in the Introduction and references, including questionable or incorrect citations, misinterpretation of key data, and reliance on sources that are difficult to verify or not clearly peer-reviewed. It is not clear whether the thematic structure was derived inductively from the data or guided a priori by the One Health framework. This ambiguity affects the epistemological status of the findings. Relatedly, the description of the analytic approach, saturation, and the pilot study would benefit from greater transparency. The Results and Discussion sections are generally well aligned, but some findings introduced in the Results

are only briefly taken up in the Discussion, while others are used primarily to support broader AMR arguments. In this respect, the Introduction and Discussion would benefit from more consistently foregrounding caregivers and children to better align with the stated focus of the study. Several issues in presentation and reporting (e.g., Table 1, quotation formatting, terminology, the language, and the Conclusions section) should be addressed to improve clarity and coherence.

Specific comments

Introduction

All the references and many of the claims at the beginning of the Introduction are questionable (p.4, lines 5-17):

- The authors may consider citing the 2016 O'Neill report, if they really want, rather than the press release about it.
- I believe reference 2 does not exist in the current form (see the 'References' section of my report).
- Reference 3 is hard to find (I did not find it). Moreover, it seems to be a non-peer-reviewed report.
- The claim at p.4, lines 11-13, that India "together with Brazil, China, Russia, and South Africa, accounts for 76% of global antibiotic consumption", is problematic at many levels: 1) it is supposed to be supported by reference 4 (for the multiple problems about reference 4, see the 'References' section of my report), but the information reported comes from a previous work, namely Van Boeckel TP, Gandra S, Ashok A, Caudron Q, Grenfell BT, Levin SA, et al. Global antibiotic consumption 2000 to 2010: An analysis of national pharmaceutical sales data. Lancet Infect Dis. 2014;14:742–50. doi: 10.1016/S1473-3099(14)70780-7, which should be used as reference here; 2) the authors wrongly reported the content of Van Boeckel et al, in that the named countries were not to be accounted for 76% of the global antibiotic consumption, but that is the share of the increase in global antibiotic consumption between 2000 and 2010 attributable to those countries; 3) the misinterpreted data refer to a paper from 12 years ago.
- It is odd to claim at p.4, line 7 that AMR "could lead to 10 million deaths each year by 2050", and then claim at lines 13-17 that "Recent research indicates South Asian region, likely to experience severe morbidity and mortality due to AMR, with an estimated 11.8

million deaths directly attributable to AMR by 2050." By the way, from what I can see, the cited document is a piece of journalism.

Please note that WHO's Global Action Plan on AMR appears under two different reference numbers (22 and 40), suggesting that the authors may have referred to the same material (see the 'References' section of my report for specific issues). If so, please fix it.

The manuscript outlines three main drivers of AMR in India (OTC antibiotic access, use in food animals and agriculture, and environmental contamination). It is not clear whether this categorisation is drawn from existing frameworks or whether these categories were identified by the authors.

Methodology

The manuscript refers to the ONE-HEART cohort without explaining what it is. A brief description, a reference or a link to a protocol or project description would help readers understand the sampling context.

The authors mention reaching data saturation as the basis for stopping data collection. It would be helpful to briefly clarify how saturation was assessed (e.g., during data collection or analysis, and at what level) and to what type of saturation they refer. The authors may consider adding a reference to clarify these and other aspects of saturation in the methodology and strengths and limitations sections.

P.6, lines 36-38, it is stated that a pilot was conducted. However, no further information is provided (e.g. participant characteristics, sample size, whether pilot data were included in the analysis, or what changes resulted from piloting).

P.6, line 58: "Each participant was assigned a unique identification number to ensure anonymity." The sentence may be misleading as it may suggest anonymisation. I think assigning unique identification numbers supports confidentiality through pseudonymisation rather than full anonymity. The authors may wish to adjust the wording accordingly.

Unfortunately, I found the 'Data Analysis' section problematic. First, it is not clear how the authors understand and operationalise their thematic analysis in relation to the One Health

framework. The analysis is described as proceeding from data segments to sub-codes, codes, categories, sub-themes, and themes, thus an inductive process. At the same time, the manuscript states that the three major themes were agreed upon “based on the One Health approach”. Were these domains (human, animal, environment) used as an a priori analytic framework guiding theme development, or did they emerge inductively from the data and were subsequently interpreted through a One Health lens (and then maybe more adequate for the Discussion)? Making this distinction explicit is important to understand the epistemological status of the findings.

Second, the manuscript refers to a ‘phenomenological approach’ (without any references that could help) in the study design, yet the analysis is described in terms of thematic coding and categorisation. A clarification would be needed about how the phenomenological orientation informed the study and the analysis.

Third, while the involvement of multiple researchers in coding and discussion is noted, a brief clarification of how analytic agreement was reached (e.g. independent coding, discussion of discrepancies, development of a shared codebook) would be informative.

Results

About Table 1: 1) the arrows/inequality symbols used for monthly household income categories seem to be incorrectly oriented (>10,000 and 30,000<). Also age (60<); 2) the occupational categories ‘Job’ and ‘Labour’ are unclear and potentially overlapping.

In the presentation of quotations, explanatory text appears within quotation marks (e.g. ‘Reason for not consuming boiled water’), which blurs the distinction between participant speech and author interpretation/explanation. Although those explanations are presented within parentheses, consider moving such clarifications outside the quotation or presenting them in a way that clearly distinguishes between the authors and the participants.

Discussion

Overall, the Discussion section engages with the main findings; however, some results introduced in the Results section are only briefly addressed. For example, caregivers’ descriptions of common childhood illnesses and their causal interpretations, as well as the implicitly gendered nature of caregiving and decision-making, receive limited analytical attention in the Discussion.

P.20, Lines 19-27: “In these communities, caregivers overwhelmingly resorted to home-based remedies, and indigenous physiotherapies such as Thokkanam for initial symptom relief, reflecting deeply rooted cultural practices. The influence of spiritual beliefs in health decision-making, employed to treat common childhood illnesses such as fever and diarrhoea, is underscored[42]”. I somehow reacted to a regulated, science-based medical profession/term, such as physiotherapy, being used to describe traditional practices like Thokkanam, but I admit my ignorance and leave it to the authors to assess whether this is the right wording. However, my main point here is the use of the reference 42: there is no comparison/consideration or any trace of ‘discussion’ justifying the use of reference 42.

P.20, Lines 30-36: “Alarmingly, participants described reusing previous prescriptions to procure antibiotics without medical consultation. This practice is strengthened by studies in both urban and rural Indian contexts, where over-the-counter sales and informal distribution channels persist despite regulations.” The sentence linking participants’ reuse of previous prescriptions to existing studies on OTC antibiotic sales is understandable, but the phrasing is unclear. Studies do not ‘strengthen’ the practice itself; rather, they document the persistence of regulatory and distributional conditions that enable it.

Conclusions

I understand that there are many different traditions, but the Conclusion section merely summarises the results. Please consider expanding it to briefly articulate the study contribution beyond description, for example, by highlighting what these findings imply for AMR-related interventions, potential directions for policy, practice, stewardship efforts, or future research within a One Health framework.

References

Reference 2 (Bryan-Wilson, J. No time to wait: Securing the future from drug resistant infections. Artforum International 54, 113–114 (2016)) may be problematic. The title seems to refer to a 2019 IACG’s report. I cannot find the document in Artforum International, which, to my understanding, is an art magazine.

Reference 3 (Vipul Patil. Antibiotic Market Analysis and Forecast: 20225–2032. Coherent Market Insights (2025)). I assume there is a typo (20225). More troublesome is the fact that I cannot find any trace of it.

References 4 and 8 (Balachandra, S. S. Antimicrobial resistance (AMR) at the community level: An urban and rural case study from Karnataka. Jouranl of family Medicine and Primary Care (2021)) are identical. Moreover, Balachandra is not the only author and there are missing information about volume, issue etc.

Reference 22 (Chan, M. Global Action Plan on Antimicrobial Resistance. World Health Organization (2017)) cannot be found in the present form. An URL would have helped. I wonder if the authors simply refer to the content at reference 40 or some other material (I may assume from the former WHO Director-General Margaret Chan).

References 59 and 63 are identical.

Reference 66 (Thomas P. Van Boeckel. Reducing anitimicrobial use in food animals. 6 (2015)) is not properly reported (list of authors, year…). Please check.

About reference 68, are the authors sure that they want to use a presentation as a reference in an article? I mean, I have no doubts about its quality, but maybe there are more informative alternatives. Moreover, it is not a preprint.

**Reviewer**	**2**
**Name**	**Albur, Mahableshwar**
**Affiliation**	**North Bristol NHS Trust, Microbiology**
**Date**	**08-Feb-2026**
**COI**	**None**

This study is conducted by public health authoroties, Dept of health funded research organisation (ICMR) from Governament of India. It shows a glimpse of the real world perception of the rural community (people) in the heart of the global AMR challenge. The authours have set a clear objectives of the study and expected / percieved limitations. They have followed the the standard COREQ frame work of writing a qualitative research. This study sample size has reasonable number of particpants with diverse backgrounds in terms gender & socio-economic factors. The study has looked at all the three domains of One health. The illustration of one health domains of the study findings in figure 1 is very commendable.

I would like to make few comments with an aim to improve some of the short comings.

1. It seems like the focus group discussions were very open and lasted 40 minutes or so. This could have led to only the extrovert type of individuals doing most the talking and the "illiterate" or shy individuals not raising their view. How was the Focus group meeting made more balanced or equpoise? How was the input from all the 77 memebers represnted in the findings.

2. The study methodology had prompts to the focus group, but did not use specific questionaire to address all the domains. Given the shorter time allocated to the focus group (<1 hr) the discussion could have been skewed by one particular point rather than equal opportunities for all the domains of one health. How was this element addressed by the researchers or interviewers of the focus group?

3. I acknowledge the lingustic challenges and diversity of the cohort of the focus groups. Still, a semiquantitative questionnare (in addition to the open ended interview)

could have given some weightage the problems & solutions to the challenges of the care givers.

4. Most often children spend time in school than at home. why were teachers not part of the focus group (at least some of them)

Minor comments:

1. Page 4 sentences 5-7- The number of attributable deaths due to AMR is more than a million.

https://www.thelancet.com/journals/lancet/article/PIIS0140-6736(24)01867-1/fulltext

2. some of the paragraphs in the introduction section (e.g. Page 5 senstences 8- 20) and discussion section could have been simplified by dividing the sentances into short and simple.

VERSION 1 - AUTHOR RESPONSE

Reviewer: 1
Dr. Mirko Ancillotti, Uppsala University

General comments

I am thankful to the editors and the authors for the opportunity to read and comment on this manuscript. The study design and data collection appear broadly appropriate, and the empirical material has the potential to make a valuable contribution to the literature. However, in my opinion, some issues need to be addressed before the manuscript can be considered for publication. There are substantial problems in the Introduction and

references, including questionable or incorrect citations, misinterpretation of key data, and reliance on sources that are difficult to verify or not clearly peer-reviewed. It is not clear whether the thematic structure was derived inductively from the data or guided a priori by the One Health framework. This ambiguity affects the epistemological status of the findings. Relatedly, the description of the analytic approach, saturation, and the pilot study would benefit from greater transparency. The Results and Discussion sections are generally well aligned, but some findings introduced in the Results are only briefly taken up in the Discussion, while others are used primarily to support broader AMR arguments. In this respect, the Introduction and Discussion would benefit from more consistently foregrounding caregivers and children to better align with the stated focus of the study. Several issues in presentation and reporting (e.g., Table 1, quotation formatting, terminology, the language, and the Conclusions section) should be addressed to improve clarity and coherence.

We thank the reviewer for the careful reading of the manuscript and for highlighting these important points. The comments and suggestions have been carefully considered, and the manuscript has been substantially revised accordingly. In the subsequent responses, we address each of the reviewer's comments and describe the corresponding revisions made in the manuscript.

Specific comments

Introduction

All the references and many of the claims at the beginning of the Introduction are questionable (p.4, lines 5-17):

•The authors may consider citing the 2016 O'Neill report, if they really want, rather than the press release about it.

• I believe reference 2 does not exist in the current form (see the 'References' section of my report).

• Reference 3 is hard to find (I did not find it). Moreover, it seems to be a non-peer reviewed report.

• The claim at p.4, lines 11-13, that India "together with Brazil, China, Russia, and South Africa, accounts for 76% of global antibiotic consumption", is problematic at many levels: 1) it is supposed to be supported by reference 4 (for the multiple problems about reference 4, see the 'References' section of my report), but the information reported comes from a previous work, namely Van Boeckel TP, Gandra S, Ashok A, Caudron Q, Grenfell BT, Levin SA, et al. Global antibiotic consumption 2000 to 2010: An analysis of national pharmaceutical sales data. Lancet Infect Dis. 2014;14:742–50. doi: 10.1016/S1473-3099(14)70780-7, which should be used as reference here; 2) the authors wrongly reported the content of Van Boeckel et al, in that the named countries were not to be accounted for 76% of the global antibiotic consumption, but that is the share of the increase in global antibiotic consumption between 2000 and 2010

attributable to those countries; 3) the misinterpreted data refer to a paper from 12 years ago.

We thank the reviewer for the careful evaluation of the references and for highlighting these important issues. In response to these comments, we have substantially revised the Introduction section. Specifically, we have rechecked all references and rewritten the opening paragraphs to ensure that each statement is supported by appropriate and verifiable sources.

Where necessary, incorrect or unclear references have been removed and replaced with more appropriate peer-reviewed sources. In particular, we have replaced the previously cited source for global antibiotic consumption with the study by Van Boeckel et al. ***(2014)*** *as suggested by the reviewer. The text has also been revised to correctly reflect the findings of that study, clarifying that Brazil, Russia, India, China, and South Africa accounted for 76% of the increase in global antibiotic consumption between 2000 and 2010, rather than 76% of total global consumption.*

In addition, we have also reviewed the references related to global AMR burden by including more relevant references and removed sources that were not appropriate or difficult to verify. Overall, the Introduction has been rewritten to improve clarity, accuracy, and consistency of citations, and the revised version now includes the recommended and relevant references.

• It is odd to claim at p.4, line 7 that AMR "could lead to 10 million deaths each year by 2050", and then claim at lines 13-17 that "Recent research indicates South Asian region, likely to experience severe morbidity and mortality due to AMR, with an estimated 11.8 million deaths directly attributable to AMR by 2050." By the way, from what I can see, the cited document is a piece of journalism.

The Introduction has been revised. The revised text now uses widely cited global estimates from recognised reports and peer-reviewed literature to ensure consistency.

Please note that WHO's Global Action Plan on AMR appears under two different reference numbers (22 and 40), suggesting that the authors may have referred to the same material (see the 'References' section of my report for specific issues). If so, please fix it.

The duplicate citation has been corrected and the reference list has been updated accordingly.

The manuscript outlines three main drivers of AMR in India (OTC antibiotic access, use in food animals and agriculture, and environmental contamination). It is not clear whether this categorisation is drawn from existing frameworks or whether these categories were identified by the authors.

The text has been revised to clarify that the discussion of these drivers of AMR is informed by existing literature rather than representing a formal analytical framework developed by the authors. Relevant references have been added/updated to support this.

Methodology

The manuscript refers to the ONE-HEART cohort without explaining what it is. A brief description, a reference or a link to a protocol or project description would help readers understand the sampling context.

We thank the reviewer for this helpful suggestion. A brief description of the ***ONE-HEART cohort study****, including its objectives and sampling context, has now been added to the Methods section to provide clarity to readers. As a protocol or project webpage is not currently available, we have included a short description of the main study within the manuscript.*

The authors mention reaching data saturation as the basis for stopping data collection. It would be helpful to briefly clarify how saturation was assessed (e.g., during data collection or analysis, and at what level) and to what type of saturation they refer. The authors may consider adding a reference to clarify these and other aspects of saturation in the methodology and strengths and limitations sections.

We thank the reviewer for this helpful suggestion. We have now clarified in the Methods section that thematic saturation was used as the criterion for stopping data collection. Saturation was assessed iteratively during data collection and preliminary analysis through regular discussions among the research team after each set of focus group discussions, and data collection was discontinued when no new codes or themes emerged. We have also added a brief clarification in the Strengths and Limitations section noting that saturation was assessed at the thematic level within the context of the study setting. In addition, methodological references on saturation in qualitative research have been added to support this.

P.6, lines 36-38, it is stated that a pilot was conducted. However, no further information is provided (e.g. participant characteristics, sample size, whether pilot data were included in the analysis, or what changes resulted from piloting).

We have clarified the pilot testing in the Methods section (Data Collection). A pilot focus group discussion was conducted with five female caregivers aged 23–27 years from the study area who had characteristics similar to the study population. The pilot assessed the clarity and contextual suitability of the topic guide. Participants responded more readily to questions on children's health-seeking practices than to those on One Health and environmental aspects. Based on this, the guide was revised to make these questions more general. As participants met the inclusion criteria, pilot data were included in the final analysis.

P.6, line 58: "Each participant was assigned a unique identification number to ensure anonymity." The sentence may be misleading as it may suggest anonymisation. I think assigning unique identification numbers supports confidentiality through

pseudonymisation rather than full anonymity. The authors may wish to adjust the wording accordingly.

The wording in the Methods section has been revised to more accurately reflect the procedure used. The sentence now reads: "Each participant was assigned a unique identification number to maintain confidentiality, with identifiers replaced by codes during transcription and analysis." This change has been made in the revised manuscript.

Unfortunately, I found the 'Data Analysis' section problematic. First, it is not clear how the authors understand and operationalise their thematic analysis in relation to the One Health framework. The analysis is described as proceeding from data segments to sub-codes, codes, categories, sub-themes, and themes, thus an inductive process. At the same time, the manuscript states that the three major themes were agreed upon "based on the One Health approach". Were these domains (human, animal, environment) used as an a priori analytic framework guiding theme development, or did they emerge inductively from the data and were subsequently interpreted through a One Health lens (and then maybe more adequate for the Discussion)? Making this distinction explicit is important to understand the epistemological status of the findings.

Thank you for the suggestion to improve the methodological clarity of the manuscript. We have revised the data analysis section as suggested.

Second, the manuscript refers to a 'phenomenological approach' (without any references that could help) in the study design, yet the analysis is described in terms of thematic coding and categorisation. A clarification would be needed about how the phenomenological orientation informed the study and the analysis.

Thank you for this important observation. We have revised the Methods section to clarify how the phenomenological orientation informed the study design and analysis. The study was conceptually informed by a phenomenological approach to explore how caregivers interpret antimicrobial resistance based on their lived experiences and everyday practices within their community context.

During the focus group discussions, facilitators encouraged participants to openly share their perspectives while attempting to minimise preconceived assumptions, allowing participants' experiences and viewpoints to guide the discussion. For the analysis, we employed an inductive thematic analysis approach to systematically code and organise the qualitative data into themes derived from participants' meanings and narratives rather than predefined theoretical categories.

The revised text also clarifies that the study explores how caregiver practices intersect with human, animal, and environmental contexts in rural settings, highlighting how these experiences shape antimicrobial use beyond the household. Relevant methodological references have been added to support this description.

"The analytic agreement was achieved through independent coding by two researchers, followed by the iterative development of a structured codebook. The codebook was

refined through regular discussions until consensus was reached. In cases of disagreement, a third author reviewed the data and provided input to finalise coding decisions."

Thank you for this helpful suggestion. We have revised the Methods section to clarify how analytic agreement was achieved during the coding process. The revised text now reads:

Results

About Table 1: 1) the arrows/inequality symbols used for monthly household income categories seem to be incorrectly oriented (>10,000 and 30,000 Also age (60<);

2) the occupational categories 'Job' and 'Labour' are unclear and potentially overlapping

We thank the reviewer for carefully examining Table 1. Suggested corrections have been incorporated into the revised ***Table 1*** *in the manuscript.*

In the presentation of quotations, explanatory text appears within quotation marks (e.g. 'Reason for not consuming boiled water'), which blurs the distinction between participant speech and author interpretation/explanation. Although those explanations are presented within parentheses, consider moving such clarifications outside the quotation or presenting them in a way that clearly distinguishes between the authors and the participants.

Suggested corrections have been incorporated into the revised ***Table 1*** *in the manuscript.*

Discussion

Overall, the Discussion section engages with the main findings; however, some results introduced in the Results section are only briefly addressed. For example, caregivers' descriptions of common childhood illnesses and their causal interpretations, as well as the implicitly gendered nature of caregiving and decision-making, receive limited analytical attention in the Discussion.

P.20, Lines 19-27: "In these communities, caregivers overwhelmingly resorted to home-based remedies, and indigenous physiotherapies such as Thokkanam for initial symptom relief, reflecting deeply rooted cultural practices. The influence of spiritual beliefs in health decision making, employed to treat common childhood illnesses such as fever and diarrhoea, is underscored42". I somehow reacted to a regulated, science-based medical profession/term, such as physiotherapy, being used to describe traditional practices like Thokkanam, but I admit my ignorance and leave it to the authors to assess whether this is the right wording. However, my main point here is the use of the reference 42: there is no comparison/consideration or any trace of 'discussion' justifying the use of reference 42.

We have revised the wording in the Discussion to describe Thokkanam as a traditional therapeutic practice rather than using the term "physiotherapy". We have also expanded the paragraph to better contextualise our findings and clarify the use of reference 47 in relation to culturally informed health belief models in South India. In addition, we have included reference 38 to support the statement regarding delayed presentation to formal healthcare facilities and the potential for increased self-medication when symptoms persist..

P.20, Lines 30-36: "Alarmingly, participants described reusing previous prescriptions to procure antibiotics without medical consultation. This practice is strengthened by studies in both urban and rural Indian contexts, where over-the-counter sales and informal distribution channels persist despite regulations." The sentence linking participants' reuse of previous prescriptions to existing studies on OTC antibiotic sales is understandable, but the phrasing is unclear. Studies do not 'strengthen' the practice itself; rather, they document the persistence of regulatory and distributional conditions that enable it.

We have revised the sentences. "Furthermore, the findings explored the persistence of regulatory and distributional conditions, which enable the OTC sales of antibiotics."

Conclusions

I understand that there are many different traditions, but the Conclusion section merely summarises the results. Please consider expanding it to briefly articulate the study contribution beyond description, for example, by highlighting what these findings imply for AMR-related interventions, potential directions for policy, practice, stewardship efforts, or future research within a One Health framework

Conclusion section is revised as per suggested comments

References

Reference 2 (Bryan-Wilson, J. No time to wait: Securing the future from drug resistant infections. Artforum International 54, 113–114 (2016)) may be problematic. The title seems to refer to a 2019 IACG's report. I cannot find the document in Artforum International, which, to my understanding, is an art magazine.

We have removed this reference and add new standard references

Reference 3 (Vipul Patil. Antibiotic Market Analysis and Forecast: 20225–2032. Coherent Market Insights (2025)). I assume there is a typo (20225). More troublesome is the fact that I cannot find any trace of it.

We have removed this reference and add new standard references

References 4 and 8 (Balachandra, S. S. Antimicrobial resistance (AMR) at the community level: An urban and rural case study from Karnataka. Jouranl of family

Medicine and Primary Care (2021)) are identical. Moreover, Balachandra is not the only author and there are missing information about volume, issue etc.

We have removed this reference and add new standard references

Reference 22 (Chan, M. Global Action Plan on Antimicrobial Resistance. World Health Organization (2017)) cannot be found in the present form. An URL would have helped. I wonder if the authors simply refer to the content at reference 40 or some other material (I may assume from the former WHO Director-General Margaret Chan).

The WHO reference has been revised to cite the official Global Action Plan on Antimicrobial Resistance (2015) document, and the URL has been added. The duplicate reference has also been removed accordingly.

References 59 and 63 are identical.

The duplicate reference has been removed. The citation has been retained as reference 13: Mutua F, Sharma G, Grace D, et al. Animal health and drug use practices in India and links to antimicrobial resistance. Antimicrob Resist Infect Control. 2020;9:103.

Reference 66 (Thomas P. Van Boeckel. Reducing anitimicrobial use in food animals. 6 (2015)) is not properly reported (list of authors, year…). Please check.

The reference has been corrected and updated with complete bibliographic details. It is now cited as reference 21 in the revised manuscript: Van Boeckel TP, Brower C, Gilbert M, et al. Global trends in antimicrobial use in food animals. Proc Natl Acad Sci USA. 2015;112:5649–5654.

About reference 68, are the authors sure that they want to use a presentation as a reference in an article? I mean, I have no doubts about its quality, but maybe there are more informative alternatives. Moreover, it is not a preprint

The presentation previously cited as reference 68 has been removed from the manuscript.

Reviewer: 2
Dr. Mahableshwar Albur, North Bristol NHS Trust, North Bristol NHS Trust LRC
Comments to the Author:

This study is conducted by public health authorities, Dept of health funded research organisation (ICMR) from Government of India. It shows a glimpse of the real world perception of the rural community (people) in the heart of the global AMR challenge. The authors have set a clear objectives of the study and expected / percieved limitations. They have followed the the standard COREQ frame work of writing a qualitative research. This study sample size has reasonable number of particpants with diverse backgrounds in terms gender & socio-economic factors. The study has looked at all the three domains of One health. The illustration of one health domains of the study findings in figure 1 is very commendable.

I would like to make few comments with an aim to improve some of the short comings.

1. It seems like the focus group discussions were very open and lasted 40 minutes or so. This could have led to only the extrovert type of individuals doing most the talking and the "illiterate" or shy individuals not raising their view. How was the Focus group meeting made more balanced or equpoise? How was the input from all the 77 memebers represnted in the findings.

The focus group discussions were moderated by trained facilitators experienced in qualitative research, who actively encouraged participation from all participants. Moderators invited quieter participants to share their perspectives and ensured that no single participant dominated the discussion. In addition, participants within each FGD were relatively homogeneous in terms of community background and caregiving roles, which helped create a comfortable environment for participants to express their views openly.No fixed duration was predetermined for the FGDs; discussions continued until key topics were sufficiently explored, with most sessions lasting approximately 40–60 minutes. During analysis, transcripts from all FGDs were reviewed in full and systematically coded using thematic analysis. Codes and themes were developed based on patterns observed across all discussions rather than individual dominant voices, ensuring that the findings reflected the diversity of perspectives expressed by the participants. These clarifications have now been added to the Methods section of the revised manuscript.

2. The study methodology had prompts to the focus group, but did not use specific questionaire to address all the domains. Given the shorter time allocated to the focus group (<1 hr) the discussion could have been skewed by one particular point rather than equal opportunities for all the domains of one health. How was this element addressed by the researchers or interviewers of the focus group?

We thank the reviewer for this observation. The FGDs were conducted using a semi-structured topic guide that included prompts covering the key domains relevant to the One Health framework, including child health-seeking practices, antibiotic use, animal contact, and environmental exposures. While the discussions were allowed to flow naturally to capture participants' perspectives, moderators ensured that all major domains in the guide were addressed during each session. Probing questions and prompts were used to steer the discussion back to underexplored topics when necessary. This approach allowed flexibility for participants to share their experiences while maintaining coverage of the key thematic areas. We have clarified this point in the Methods section of the revised manuscript.

3. I acknowledge the lingustic challenges and diversity of the cohort of the focus groups. Still, a semiquantitative questionnaire (in addition to the open ended interview)

could have given some weightage the problems & solutions to the challenges of the care givers.

We agree that semi-quantitative questionnaires can help provide additional insight into the relative importance of specific challenges and potential solutions. However, the objective of this component of the study was to explore caregivers' knowledge, perceptions, and practices related to antibiotic use and antimicrobial resistance in depth, which is well suited to a qualitative approach. The use of semi-structured focus group discussions allowed participants to describe their experiences and perspectives in their own words and enabled the research team to capture contextual factors related to One Health domains that may not have emerged through structured questionnaires. We acknowledge that incorporating semi-quantitative tools could complement qualitative findings, and this approach may be considered in future studies.

4. Most often children spend time in school than at home. why were teachers not part of the focus group (at least some of them)

We agree that teachers can play an important role in shaping children's behaviours and could provide useful perspectives on antibiotic use and hygiene practices. However, the present qualitative component was designed to focus specifically on primary caregivers, as they are the main decision-makers regarding children's healthcare, antibiotic use, household hygiene practices, animal care, and waste management within the home environment. For this reason, participants were purposively selected from caregivers within the ONE-HEART cohort households. While including teachers could provide additional insights, this was beyond the scope of the current study. We acknowledge this as a potential area for future research exploring school-based perspectives on AMR awareness and behaviours.

Minor comments:

1. Page 4 sentences 5-7- The number of attributable deaths due to AMR is more than a million.

https://www.thelancet.com/journals/lancet/article/PIIS0140-6736(24)01867-1/fulltext

We have revised the figures

2. some of the paragraphs in the introduction section (e.g. Page 5 senstences 8- 20) and discussion section could have been simplified by dividing the sentances into short and simple.

The relevant paragraphs in both the Introduction and Discussion sections have been revised to improve readability. Longer sentences have been simplified and divided into shorter statements while retaining the original meaning and scientific content.

VERSION 2 - REVIEW

**Reviewer**	**1**
**Name**	**Ancillotti, Mirko**
**Affiliation**	**Uppsala University**
**Date**	**01-Apr-2026**
**COI**	

I thank the author for addressing my comments, and I am happy to see that the article has improved significantly.

I remain somewhat uncertain about how the analysis was conducted in relation to the One Health framework, and I wonder whether the authors could clarify this further. As I understand it, the manuscript currently suggests both that the analysis was inductive and that the human, animal, and environmental domains were used to guide theme development. I may, of course, be misunderstanding the analytic process, so I would be grateful if the authors could make this point more explicit. For example, was the analysis primarily inductive, with codes and categories developed from the data first and the findings subsequently interpreted through a One Health lens? Or was it rather the case that the human, animal, and environmental domains were used from the outset as an analytic framework within which codes and themes were organised? If the approach combined inductive coding with a more deductive or framework-guided stage, it would help to state that directly.

References need further attention. A few examples:

• Ref 31. "ET RM. Epidemiology of antibiotic resistance in children in low- and middle-income countries." It is unverifiable, lacks authorship, and omits many other details.

• Ref 20. To my knowledge, the WHO’s report has no DOI. The authors seem to refer to an editorial or similar piece discussing the report.

• Ref 53. I leave it to the journal to assess the authority and practices of the Journal of Namibian Studies. Nonetheless, or in addition, it is odd that the DOI of a paper published in 2023 is still pending.

• Ref 54. As for ref 53, I leave it to the journal to assess the authority and practices of the International Journal of Scientific Research and Technology. Moreover, the DOI is that of another article. The list of authors is incomplete.

**Reviewer**	**2**
**Name**	**Albur, Mahableshwar**
**Affiliation**	**North Bristol NHS Trust, Microbiology**

Date **17-Mar-2026**

COI

I am satisfied with explanations for all of my commments as well as by other reviwers.

VERSION 2 - AUTHOR RESPONSE

Reviewer 2: *"I am satisfied with explanations for all of my comments as well as by other reviewers."*
Response:
We sincerely thank the reviewer for their positive feedback and appreciation of our revisions.

Reviewer : ***I thank the author for addressing my comments, and I am happy to see that the article has improved significantly. I remain somewhat uncertain about how the analysis was conducted in relation to the One Health framework, and I wonder whether the authors could clarify this further. As I understand it, the manuscript currently suggests both that the analysis was inductive and that the human, animal, and environmental domains were used to guide theme development. I may, of course, be misunderstanding the analytic process, so I would be grateful if the authors could make this point more explicit. For example, was the analysis primarily inductive, with codes and categories developed from the data first and the findings subsequently interpreted through a One Health lens? Or was it rather the case that the human, animal, and environmental domains were used from the outset as an analytic framework within which codes and themes were organised? If the approach combined inductive coding with a more deductive or framework-guided stage, it would help to state that directly.***

Response: We thank the reviewer for this insightful comments, which has helped improve the clarity of our manuscript. We have now clarified the analytic approach in the Methods section. In brief, the analysis followed a **hybrid approach combining inductive and deductive elements**. Initial coding was conducted inductively, allowing themes to emerge directly from the data without imposing predefined categories. Subsequently, these themes were interpreted and organised using the One Health framework, aligning them within the human, animal, and environmental domains. This clarification has now been incorporated into the revised manuscript.

Reference Corrections
Ref 31. "ET RM. Epidemiology of antibiotic resistance in children in low- and middle-income countries." It is unverifiable, lacks authorship, and omits many other details.

Added more relevant recent, peer-reviewed study on antimicrobial resistance among children in low- and middle-income countries: Okumu NO, Muloi DM, Moodley A, et al. (2025). *Frontiers in Microbiology*. https://doi.org/10.3389/fmicb.2025.1539160

Ref 20. To my knowledge, the WHO's report has no DOI. The authors seem to refer to an editorial or similar piece discussing the report.

The incorrect DOI has been removed. The WHO report is now cited using the appropriate institutional format with the official URL.

Ref 53. I leave it to the journal to assess the authority and practices of the Journal of Namibian Studies. Nonetheless, or in addition, it is odd that the DOI of a paper published in 2023 is still pending.

This reference has been replaced with a more authoritative, peer-reviewed source:
Pradhan MR, De P. (2025). *BMC Health Services Research*, 25:323.
https://doi.org/10.1186/s12913-025-12463-9

Ref 54. As for ref 53, I leave it to the journal to assess the authority and practices of the International Journal of Scientific Research and Technology. Moreover, the DOI is that of another article. The list of authors is incomplete.

This reference has been replaced with a high-quality, peer-reviewed publication with complete authorship and correct DOI: Kruk ME, Gage AD, Arsenault C, et al. (2018). *The Lancet Global Health*, 6(11): e1196–e1252. https://doi.org/10.1016/S2214-109X(18)30386-3
